# Psychosocial burden of localised cutaneous Leishmaniasis: a scoping review

**DOI:** 10.1186/s12889-018-5260-9

**Published:** 2018-03-15

**Authors:** Issam Bennis, Vincent De Brouwere, Zakaria Belrhiti, Hamid Sahibi, Marleen Boelaert

**Affiliations:** 1 National School of Public Health, Ministry of Health, Lemfedel Cherkaoui Street, Madinat Al Irfane, 10000 Rabat, Morocco; 2 Department of Public Health, Institute of Tropical Medicine, Antwerp, Belgium; 3 Department of Biomedical Sciences, Faculty of Pharmaceutical, Biomedical and Veterinary Sciences, University of Antwerp, Antwerp, Belgium; 4 Vrije Universiteit Brussel, Rabat, Morocco; 5 Department of Pathology and Veterinary Public Health, Hassan II Agronomy and Veterinary Institute, Rabat, Morocco

**Keywords:** Cutaneous leishmaniasis, Cicatrix, Psychology, Social stigma, Quality of life

## Abstract

**Background:**

Cutaneous Leishmaniasis (CL) is a parasitic skin disease, linked to poverty, and belonging to the group of Neglected Tropical Diseases. Depending on the severity, the type of lesions or scars, and the context, CL can lead to self- and social stigma influencing the quality of life and psychological well-being of the patient. This dimension is, however, little documented for the most common, localized form of cutaneous leishmaniasis (LCL).

We aimed to describe the current knowledge on the psychological burden and the stigma related to LCL.

**Methods:**

The population of interest for this scoping review are patients or their relatives with localized LCL or related scars.

We searched the electronic databases PubMed, Web of Knowledge, PsycINFO, POPLINE, Cochrane Library, Science Direct, Global Health, and LILACS, for articles written in Arabic, English, French, Dutch, Portuguese, or Spanish, and published until the end of August 2017.

**Results:**

From 2485 initial records, 15 papers met our inclusion criteria. Dermatology life quality index was the most frequent used scale to assess LCL psychological impact in quantitative studies. Six qualitative studies used individual interviews and/or focus groups discussions to explore the psychological and/or the social burden of this disease. Quantitative assessments using standard scales as well as qualitative research asserts that LCL is a source of psychological suffering, stigmatization, and decreased quality of life (QoL).

**Conclusion:**

Most studies showed that LCL has a significant negative effect on the QoL and mental health. However, the fact that the psychosocial burden generated by LCL is time-dependent makes it hard to measure. We recommend to develop a more specific and validated assessment scale to appreciate the full burden of this disease and enhance comparability of findings.

**Electronic supplementary material:**

The online version of this article (10.1186/s12889-018-5260-9) contains supplementary material, which is available to authorized users.

## Background

Cutaneous leishmaniasis (CL), a neglected tropical disease (NTD), is a parasitic infection of the skin characterized by slowly healing ulcers that can leave indelible scars. There are various clinical presentations of CL: localized, diffuse, disseminated and mucocutaneous CL.

The global mean age-standardized disability-adjusted life years (DALYs) lost by CL were estimated at 0.58 per 100,000 people [1]. However, several authors have criticized the way the global burden of disease tools, such as DALYs, have quantified the burden of CL. A recent viewpoint drew attention to the fact that only active forms of CL are included in DALY calculations. It suggests to consider also the psychological impact linked to the disfiguring scars of CL [2]. Moreover, the same authors proposed to multiply by ten the actual burden of CL, to take into account the effect of CL scars [2]. Under this scenario, the global number of people living with LCL scars is estimated at 40 million. Such claims call for a higher prioritization and resource allocation to CL [2].

Localized CL (LCL) is by far the most common presentation, usually as a single papular or nodular skin lesion that progressively ulcerates and self-heals within a few months [3].

In 2013, LCL was primarily reported from 15 countries: Afghanistan, Algeria, Brazil, Colombia, Honduras, Iran, Morocco, Nicaragua, Pakistan, Peru, Saudi Arabia, Syrian Arab Republic, Tunisia, Turkey and Yemen [4].

LCL is often presented in the literature as a dermatological disorder that is “*associated with stigma*” and psychological suffering, which is quite obvious for the severely mutilating or disseminated types of CL. Severe scar tissue from any cause (acne, burn, injury, Buruli ulcer, lymphatic filariasis, podoconiosis, a.o.) reduces the quality of life in a direct way but also through a process of social rejection and self-depreciation [5–13]. In some skin disorders, this psychosocial effect is predominant, as in vitiligo patients, who experience a reduced quality of life because of their social rejection [14–18]. The psychological distress generated by skin ailments is complex and depends on the severity and visibility of the lesions. Skin disorders are no exception to the general rule that the degree of fear inspired by a disease is mainly associated with the perception of severity and also lack of adequate treatment [19]. In the past, the typical examples were smallpox and leprosy that led to a total isolation of those affected, and more recently, Ebola, HIV/AIDS, psychiatric and mental illnesses continue to generate this stigmatizing behavior in many communities. The resulting social isolation affects a human being psychologically and this was shown in the case of the recent Ebola epidemic to have a negative impact on survival [20, 21]. However, the experience in psoriasis shows that the relationship between symptom severity and psychosocial distress reported by psoriasis patients is not straightforward [22]. Nevertheless, a systematic review showed that clinical depression was 1.5 times more frequent in psoriasis patients than in non-affected peers [23].

In the literature on the psychosocial burden of disease, stigma is a central concept. Mostly stigma manifests itself in discriminatory attitudes against those who are different and is related both to the conception of the ‘other’ as well as the ‘me’ [24]. This conceptual complexity makes stigma difficult to determine. Goffman defines stigma as: ‘*The phenomenon whereby an individual with an attribute which is deeply discredited by his/her society is rejected as a result of the attribute. Stigma is a process by which the reaction of others spoils normal identity’* [19, 25]. Other authors define stigma as a social process that is characterized by exclusion, rejection, and blame resulting from the anticipated perception or expression of an adverse social judgment about a person or a group [26, 27]. This judgment can be related to the mental disorder, ethnicity or physical handicap of the stigmatized persons [25]. Stigmatisation also affects people who are not perceived as a threat to others but who present a disfigured body [28, 29]. The judgments made are often the result of comparisons to a perfect, idealized body image [30]. Often shaped on the pictures of ‘celebrities’ as the ideal social appearance, this idealized body image increases the pressure on those who appear different, pushing some of them to seek cosmetic surgery [31]. Stigma is also typically attached to those conditions in which people are blamed for their appearance [32]. The social rejection generates suffering, as the stigmatized person interiorizes what others think of her/him, leading to loss of self-esteem and what is called “self-stigma” [33]. Once the individual perceives the stigma, it can affect his/her quality of life [15]. The more general concept of “stigma” has, therefore, two dimensions: how others relate to the stigmatized person and how that person relates to him/herself. Henceforward in this text, we distinguish these two dimensions based on the model presented previously by Deacon [34] and adapted by Weiss in 2008 [27]:

1. Social stigma (public stigma) occurs when society actively adopts negative attitudes of rejection or exclusion against the stigmatized who undergo inequitable or discriminatory treatment. Enacted stigma, which is about passive discrimination, is one expression of social stigma. Even if some people disagree with the stigmatizing behaviors of others, they often remain passive about it, without trying to stop it.
2. Self-stigma is an internalized mechanism in the stigmatized person, who is anticipating the social rejection or is adapting to a stigmatizing environment. In this category, we can include: felt stigma, experienced stigma, internalized stigma, internal stigma, anticipated stigma, imagined stigma, perceived stigma.

Then, whether the painless nodules, ulcers, and scars of LCL contribute to the global burden of CL beyond the local discomfort they generate is an open question. So far, little research has been done to underpin this general statement about LCL, nor to differentiate the burden according to CL type.

Our review aimed to describe the current evidence related to the psychosocial burden of LCL. More specifically, our objectives were to (i) explore which quantitative scales were used to assess this burden; (ii) summarize qualitatively the extent of psychosocial suffering (specifically self-stigma, social stigma, quality of life) attributable to LCL. Our research question was *“Do persons experiencing a LCL skin lesion suffer from any psychosocial burden?”*

## Methods

A scoping review is mainly based on a preliminary assessment of the amount/the extent of available research in the literature [35]. Following Arksey and O’Malley’s framework we worked through five stages, listed hereafter. Stage 1: identifying the research question; Stage 2: identifying relevant studies; Stage 3: study selection; Stage 4: charting the data; Stage 5: collating, summarizing and reporting the results [36, 37].

### Inclusion and exclusion criteria

#### Inclusion criteria

**Population**: Persons (patients or their relatives) experiencing a skin condition linked to LCL.

**Range of concepts**: LCL-related stigma and its psychological consequences in different LCL skin conditions, including LCL scars.

**Context**: All countries.

All articles discussing entirely or partially the stigma types, psychological and social consequences and quality of life, psychological or psychosocial effects related to Leishmaniasis skin condition. All types of publications were eligible: original articles, literature review, editorial, comments, correspondence, abstracts of workshop or conferences, grey literature, as available online.

#### Exclusion criteria

As listed in Additional file 1, we excluded articles for which no full text was available; books; articles targeting veterinary studies, vectors, laboratory or in-vitro research; studies describing CL epidemiology, outbreaks or trends without or with a little indirect information about psychosocial outcomes; articles exclusively targeting HIV or mental illness or other stigmatising diseases without any link with CL; articles treating only the visceral form of leishmaniasis or post dermal kala azar form or exclusively the CL mucosal or tegumentary form; articles on diagnostic methods for CL; treatment assessment or methods not targeting psychosocial interventions in CL (e.g. drug effectiveness).

### Search strategy

Eight databases were selected: PubMed, Web of Knowledge, Science Direct, PsycINFO, POPLINE, LILACS, Global Health CABI and Cochrane Library. We searched these databases from their inception until the end of August 2017 with language restriction to Arabic, English, French, Dutch, Portuguese, and Spanish.

The search in the PubMed and Web of Knowledge databases followed the three steps recommended in Peters et al. [38]. (1) Firstly, we did a limited exploratory search in PubMed combining three keywords: Cutaneous leishmaniasis AND (Stigma OR Quality of life). Then, we built a logical grid after analyzing text words contained in the title and abstract with adding the subject heading and index terms. (2) Secondly, we organized them into blocks by using the advanced search composed of MeSH terms and free synonymous words categorized into three blocks (1#Population, 2#Concept, 3#Context) which helped us to build the query question and perform the primary search strategy (as listed below). For the other databases, shorter search equations were set to find relevant references to our topic (See Additional file 1).

(3) Thirdly we performed a hand search on the reference list of the selected articles and searched for any publication by the first, second, third and last authors in those selected articles and meeting the same inclusion criteria but initially not detected by our initial search strategy. Also, we looked for overall articles that cited the ones we selected. We repeated this process iteratively until no other relevant articles were found.

### Query search (PubMed) and (web of knowledge)

This search strategy was done end of September 2016 and updated on 1st September 2017.

(((Leishmaniasis OR Dermal Leishmaniasis OR Cutaneous Leishmaniasis OR Mucocutaneous Leishmaniasis OR Diffuse Cutaneous Leishmaniasis NOT Visceral Leishmaniasis)) AND (Social problems OR Public opinion OR Social discrimination OR Social isolation OR Social exclusion OR Social distance OR Stereotyping OR Social perception OR Social conditions OR Social adjustment OR Social behavior OR Social behaviour OR Social behavior disorders OR Social environment OR Social support OR Social marketing OR Cost of illness OR Stigma* OR Public stigma OR Social Stigma OR community stigma OR Enacted stigma OR Self-stigma OR Self stigma OR Experienced stigma OR Perceived stigma OR imagined stigma OR Anticipated stigma OR Felt stigma OR Self-report OR internalized stigma OR Health related stigma OR Gender Identity OR Quality of Life OR Perception OR Rejection OR Discrimination OR Parasitic skin diseases/psychology OR Skin diseases, bacterial/psychology OR Neglected Diseases/psychology OR Psychosocial OR Psycho-social)) AND ((“1920/01/01”[PDat]: “2017/08/31”[PDat]) AND (Arabic[lang] OR English[lang] OR French[lang] OR Dutch[lang] OR German[lang] OR Spanish[lang]))

### Data collection, extraction, and analysis

Two investigators (IB, ZB) independently reviewed the titles and abstracts of all retrieved records and selected the articles for full-text review based on eligibility criteria as defined below. EndnoteX7 (Thomson Reuters 2014) was used as a reference manager software. This software served to avoid duplication, achieve the first screening of titles or titles and abstracts, download and manage full texts of selected references for a complete reading step and provide a digital backup for archiving and enhancing traceability of the search process for this review. In case of disagreement, the last author (MB) served as the tiebreaker. The other authors, all experts in the field of CL, stigma and conducting systematic reviews, rechecked the inclusion and exclusion criteria of selected articles.

**Fig. 1:**
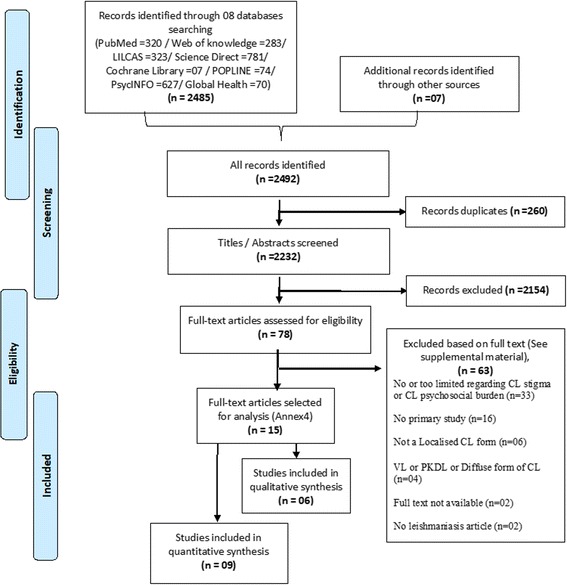
Flowchart of the number of literature searched and selected for psychosocial burden of Cutaneous Leishmaniasis

The results of the literature search are presented in a flow diagram (Fig. 1) describing the scoping review process [38]. The Additional file 1 includes more details on the search: activities performed with date, the query equation employed, and the reference list found, including justification for excluding references after full-text reading and the final references of the included articles.

We extracted the data and mapped them in an Excel sheet (Windows, Microsoft Corporation). We used a narrative synthesis approach to compile the data and examples from individual studies. We focused on how studies addressing a different aspect of the same phenomenon can be narratively summarized to provide a bigger picture of that phenomenon. Using Nvivo11 QRS International, we classified them under main conceptual categories (Additional file 1).

We followed the five steps of reporting scoping reviews and used the PRISMA flowchart for reporting the search strategy [36, 37, 39].

## Results

### Included papers

We included 15 articles matching our criteria for a full review (Table 1) [40–54]. The studies were done in nine countries: Afghanistan, Iran, Morocco, Sri Lanka, Surinam, Syria, Tunisia, and Yemen. Nine papers used quantitative research methods, including eight cross-sectional descriptive surveys and one randomized controlled trial of a psychotherapy intervention in LCL. One was based on mixed methods while five were based on qualitative research: one of them was based on focus group discussions, one was a review, and the others were based on interviews.

**Table 1:** Studies included in this review by study design, study population characteristics and sample size

Authors	Year of publication	Country of the study	Methods	Study design	Timing	Study Population	Sample size	Sex ratio (M/F)	Age range (years)	Patients with CL scars included
Al-Kamel et al.	2017	Yemen	Qualitative	Interview	May 2016	CL patients	11	0.10	12–60	Yes
Bennis et al.	2017	Morocco	Qualitative	Questionnaire	April 2015	Boarding school students	448	1.36	16–20	Yes
Chahed et al.	2016	Tunisia	Quantitative	Questionnaire survey (scale)	Not defined	Women with CL scar	41	All female	12–53	Yes
Ramdas et al.	2016	Surinam	Qualitative	Ethnography	Sep 09 to Dec 10	CL patients. General population	205CL patient & 321 people	8.3 & 1.4	20–49	Yes
Turan et al.	2015	Turkey	Quantitative	Questionnaire survey (scale)	May 11 to Apr 13	Paediatric CL patients and healthy controls	54 CL patients	1.16	7–18	No
Handjani et al.	2013	Iran	Quantitative	Questionnaire survey (scale)	2013	Relatives of dermatological cases	50 relatives	0.85	20–65	No
Vares et al.	2013	Iran	Quantitative	Questionnaire survey (scale)	Not defined	CL patients	124	0.59	16–80	Yes
Abazid et al.	2010	Syria	Quantitative	KAP-survey	Nov 06 to Oct 07	CL patients or the caregiver seeking treat	70	0.46	≈32	No
Fernando et al.	2010	Sri Lanka	Quantitative	KAP-survey	Sep 06 to Feb 08	CL patients	120	2.75	02–70	No
Nilforoushzadeh et al.	2010	Iran	Quantitative	RCT	2007	Female CL patients	2 groups of 20	All female	> 10	No
Kassi et al.	2008	Afghanistan	Qualitative	Case report	Not defined	Woman with CL scar	1	All female	28	Yes
Simsek et al.	2008	Turkey	Quantitative	Questionnaire survey (scale)	2006	Women in general population	247	All female	15–47	Yes
Reithinger et al.	2005	Afghanistan	Mixed	KAP & FGD	Oct 2002	KAP: household head; FGD: women	KAP: 252; FGD: 108	FGD All female	Not defined	Yes
Yanik et al.	2004	Turkey	Quantitative	Questionnaire survey (scale)	Sep 02 to Aug 03	CL patients, persons with CL scar, healthy controls	3 groups of 33	1:1	12–35	Yes
Reyburn et al.	2000	Afghanistan	Qualitative	FGD	Feb to Jul 1998	CL patients and unaffected spouses	8 groups of 6 to 10	1:1	≈28	Yes

### Psychosocial burden of LCL measured quantitatively

*Anxiety and depression:* Seven quantitative studies measured the psychosocial outcome on a standard scale (Table 2). Yanik et al. [41] assessed the psychological impact of LCL in three groups of patients in Turkey (LCL-active lesions, LCL-scars, and a control group). They used three different scales to quantify 1. Anxiety and depression (HAD), 2. Body Image Satisfaction (BIS), and 3. Dermatology Life Quality Index (DLQI). They concluded that anxiety and depression were frequent symptoms in patients with LCL at both the active lesion and scar stage and affected their quality of life. Patients with LCL scars had better quality of life scores than those with active lesions. Simsek et al. [43] in Turkey used the DSM classification scale of mental illness (DSM-IV) to study predictors of mental disorders in a sample of women from a rural South-eastern region in Turkey. In multivariate analysis, LCL was found to be an independent predictor of mental illness (Odds Ratio (OR) 2.15, 95% C.I. 1.25 to 7.31) at the same level as domestic violence (OR 2.03, 95% C.I. 1.17 to 4.28). In 2015, Turan et al. [50] in the same endemic area in Sanliurfa, used four scales in children and adolescents from 7 to 18 years and their parents. In the patients with LCL, depression scores were significantly higher than in the control group, and this was also the case in the group of mothers of children with LCL. No difference was found in anxiety scores.

**Table 2:** Overview of the quantitative studies included in this review based on scales questionnaire surveys

Reference	Measurement scales of psychological, social and QoL burdens	Main findings
Chahed-2016	Revised Illness Perception Questionnaire (IPQ-R), World Health Organization Quality of Life-26 (WHOQOL-26)	Large range of negative psychological consequences of LCL it is a source of constant stress and shame. Leading to social rejection and anticipation/ avoidance behaviour.
Turan-2015	The Child Depression Inventory (CDI) The State-Trait Anxiety Inventories for Children (STAIC), The Paediatric Quality of Life Inventory Parent and Child Versions (PedQL-P and C)	Depression scores were significantly higher in children and adolescents with CL compared to age-matched controls. No significant difference was found in anxiety scores. Depression scores were also higher in mother of children with LCL.
Handjani-2013	Family Dermatology Life Quality Index (FDLQI)	Family members of LCL cases face reduction in QoL, mainly due to the time they have to spent looking after the patient
Vares-2013	Dermatology Life Quality Index (DLQI)	The type of LCL lesion determines the degree of QoL reduction. Most reduced in ulcers, least in papular LCL
Nilforoushzadeh-2010	Dermatology Life Quality Index (DLQI)	The effect of adjunct psychotherapy to drug treatment on QoL was tested in a randomized controlled trial on women with LCL. QoL was improved in the intervention group.
Simsek-2008	The Turkish version of the Structured Clinical Interview for Axes I of the DSM-IV (SCID-I)	This study on mental health of women in rural Turkey identified CL as one of the main independent predictors of mental disorders
Yanik-2004	Hospital Anxiety Depression Scale (HAD), Body Image Satisfaction Scale (BIS), Dermatology Quality of Life Scale (DQL)	Depression and anxiety are higher in LCL compared to control. Impaired body satisfaction in LCL. LCL have reduced QoL compared to LCL scars and controls.

*Low quality of life:* Two studies were conducted in Iran [45, 48] using the DLQI scale to assess the QoL in LCL patients under treatment. Vares et al. [48] found that the type of LCL lesion had a significant effect on the patient’s QoL. The DLQI score in patients with papular LCL lesions was better than in those with nodular and plaque lesions. Those with ulcerated lesions had the lowest QoL score. Nilforoushzadeh et al. [45] randomly allocated 40 women with LCL active lesions to either the standard LCL treatment or standard treatment plus psychotherapy. The intervention arm showed a more pronounced improvement in DLQI score at the end of the study after 8 weeks (*p* = 0.001). Hanjdani et al. [49] used the Family DLQI scale to assess the impact of chronic skin disorders on family members. A variety of chronic skin disorders of at least one-month duration (vitiligo, pemphigus, psoriasis, and LCL) were included. There was a reduction of QoL, without a significant difference between disorders, but the sample size was small. The main problems mentioned were emotional distress, spending much time with the patient, and reactions of the others to the patient’s condition. The main complaint in families with a LCL case was about the time spent looking after the patient.

*Stigma:* In Tunisia, Chahed et al. [52] assessed the level of stigma (using the Psoriasis Life Stress Inventory and QoL (Revised Illness Perception Questionnaire and the World Health Organization QoL26) in a sample of 41 women with LCL scars and reported a wide range of psychological effects. 73% of the women had suffered social exclusion and stigmatization. They reported broken relationships, interpersonal conflicts, reduced employment opportunities, and reduced marriage prospects for men and women. LCL was a constant source of stress for the study participants. They thought their scars were degrading, and a source of shame. The “experience of social rejection” and the “anticipation and avoidance of stress” were significantly and negatively correlated with age. The younger the person, the more impact CL had in terms of stigma. QoL was correlated with the “anticipation of stress” score but negatively correlated with knowledge about CL. The more a person knew about LCL, the less likely they were to view their quality of social life negatively.

*Fear of scars:* The two other quantitative studies included in this review [46, 47] were both based on a general questionnaire targeting knowledge, attitudes, and practices of LCL patients in endemic areas from Syria and Sri Lanka. In both studies, no direct information about QoL was provided except the adverse effect of fear about LCL scars, especially on the face.

### Psychosocial burden of LCL reported qualitatively

Very recently five qualitative studies provided more information on the CL psychosocial burden from the perspective of the general population and affected patients living in LCL endemic areas (Table 3).

**Table 3:** Overview of the included studies analysed qualitatively

1st Author name, Year of publication and country of the study.	Types of stigma noticed	Types of psychological consequences related to CL	Reasons for stigma	Reasons for Psychosocial burden
Al-Kamel-2017 (Yemen)	Social stigma Aesthetic stigma Psychological stigma	Stress Depression Anxiousness Shame	Socially isolated	Feels shame. Fears of infecting their children and infecting others through food and drink. Fears of deformity, disability and death. Fear of malignancy.
Bennis-2017 (Morocco)	Self-stigma Social stigma	Large spectrum of negative psychosocial effects: Anxiety, Embarrassment, Sadness, Shame, Suicidal ideas	Social stigma (Family relationships, social contempt, fear, marriage difficulties, avoidance by others). Self-stigma (embarrassment, shame, sadness, anxiety, depression, suicidal ideas)	Emergence of psychological consequences due to perceived stigma.
Chahed-2016 (Tunisia)	Social exclusion and stigmatization	Loss of self-esteem, feelings of inferiority and the idea that the disease is an obvious social disadvantage. There was also a strong sense of shame.	The nature of stigma that patients experience is associated with certain general fears and anticipation of rejection, rather than the actual rejection that patient truly experience from society. It reduced marriage prospects for men (75%) and women (59%). ZCL had no positive effects on their lives. The consequences have rather been negative. About 73% suffered social exclusion and stigmatization. Their relationships have been broken and they face more interpersonal conflicts in society, regardless of the context (family, social or professional). The consequences were seen also in their chances of getting employment	Respondents are dissatisfied with their self-image and appearance. Scars alter women’s beauty (58%) Drugs were seen to have little or no effect, and the risk of getting the disease again is likely. The more a person looks at the world anticipating segregation and rejection, the more they perceive ZCL as a disease with harmful effects on them-The more they see it as a mysterious and incomprehensible illness, and the more they talk about suffering and emotional difficulties.
Ramdas-2016 (Surinam)	Aesthetic stigma In the literature: 1) experienced or enacted stigma; 2) anticipated, felt, or perceived stigma; and 3) internalized or self-stigma	38 CL patients who reported having experienced negative reactions related to CL expressed feeling bad about their looks. Some said they were “disgusted” by their sores, others reported feeling “shy” or “ashamed”.	Health related stigma is linked to patients’ own illness experiences’	These feelings were experienced when sores became bigger, or when they had multiple sores. The gruesome appearance of CL sores contributed to patients’ fear of the disease. Aside from a sore’s appearance, the growth of a sore, the increase in number of sores, and their visibility could also cause overt negative reactions from those in a patient’s environment.
Reyburn-2000 (Afghanistan)	Enacted stigma Feeling rejection and isolation	Some women expressed anger at the situation Felling bring shame to the whole family. One girl said that she sometimes felt suicidal.	Women tended to express feelings of rejection and isolation more frequently than men Widespread and inappropriate fear of direct or indirect transmission of CL to others. Fear of contagion and an association with dirt, low personal hygiene, sinfulness and disgust. If you commit a sin or you have done something wrong, you will get the punishment from God later on. An insulting word, “soldana” was commonly reported to be used for CL sufferers, leading to a general feeling of victimization. Some respondents were required to eat separately from the family with his/her own plate, cup, utensils and not to share a bed with healthy family-members. Women with CL often reported that they were not allowed to cook for the family. Had lost authority or felt disempowered from interacting in public or managing their affairs. It seemed that the perceived need to isolate CL sufferers from others readily developed into a more personal rejection.	One of the major concerns for young people, especially women, was their appearance and adherence to an ideal of beauty. CL was felt to seriously undermine their prospects for marriage. Both active lesions and scars were a concern, particularly on the face. Several men reported that leishmaniasis caused problems in taking their ablutions and staying ritually clean throughout the prayer. They felt that others saw them as unclean and they were deterred from praying at the mosque.

*Expectation of stigmatisation:* The most important effects were linked to LCL patients holding scars on visible parts of their body, specifically on women faces’ more than men. Chahed et al. [52] reported feelings of inferiority and the idea that the disease is equal to an apparent social disadvantage. Fifty percent of all women with scars said scars alter their beauty.

*Perception of changed body image:* The concept of loss of beauty has been a frequent feature of all these studies, especially for young bachelors. The impact of unwanted changes in body image is considered as a curse or bad luck that will diminish the marriage opportunities of the person suffering from LCL scar.

*Self stigma:* A broad spectrum of psychosocial suffering was reported such as being shy, ashamed, stressed, anxious, depressed. Feeling embarrassment, sadness, suicidal thoughts and decrease of self-concept (self-confidence, self-esteem, self-contempt, self-awareness) lead patients to perceive or interpret negatively some direct or indirect reactions from partners, relatives, and the general population.

Some women affected by LCL try to hide their lesions from their partner and look for more permanent solutions by surgical repair or aesthetic surgery. As Chahed et al. [52] have pointed out, the extent of the stigma that patients experience is associated with their anticipation of rejection.

*Social stigma:* From emotional isolation and emotional distress to firm social rejection and discrimination within the same household, with friends or/and workmates, or abroad family visitors. Some affected people felt their chances to find employment, studies or marriage jeopardized. However, simultaneously, the perceived stigma which was expressed or enacted by the neighborhood led to psychological consequences as well. In Afghanistan [40], women with LCL frequently felt rejected. As LCL was relatively new in Kabul, there was a widespread misconception about its origin, with fear of contagion and an association of LCL with dirt and low personal hygiene. People attributed LCL to “*the fault and sin carried by strangers living in neighboring provinces or cities.”* Such misconceptions raise the level of rejection and social exclusion.

*Mitigating factors of stigma (Resistance to stigma):* The tendency to isolate LCL patients from others contributes to more self-stigma. The patients try to hide or mask the lesion, by using homemade or commercial makeup, or by wearing the traditional veil. The qualitative studies also reported that LCL being for some people only a minor source of suffering, for the following reasons. Firstly, people living in rural areas consider it quite normal to have scars because of the forestry and agriculture activities which expose them to several abrasions and wounds. Thus, an ulcer that self-heals over time is not a major problem for them. Such scars are sometimes even considered as a mark of belonging and membership to a community, even as a mark of glory and pride. Secondly, religious people tend to accept this kind of ailments, as a submission to the will of the Almighty. Finally, old people have less fear about aesthetic outcomes than younger people.

## Discussion

The main finding of our review is that LCL indeed generates psychological suffering and mental ill-health, as shown in several countries. LCL leads to social stigma that eventually causes self-stigmatization, which amplifies the feelings of fear, anxiety, and depression in those affected. Extreme self-isolation and self-contempt can sometimes even lead to suicidal ideation. Moreover, young single women with facial scars face the most substantial psychosocial impact of LCL [40, 52, 54, 55]. Of course, there is a contrast with the ill-health generated by the more severe diffuse, disseminated and mutilating mucocutaneous CL forms, which can even be life-threatening. Getnet’s work in Ethiopia [56] and in Latin America [57–61] showed the psychosocial burden of these non-localized CL types for which the prognosis is much more severe than that of LCL.

**Fig. 2:**
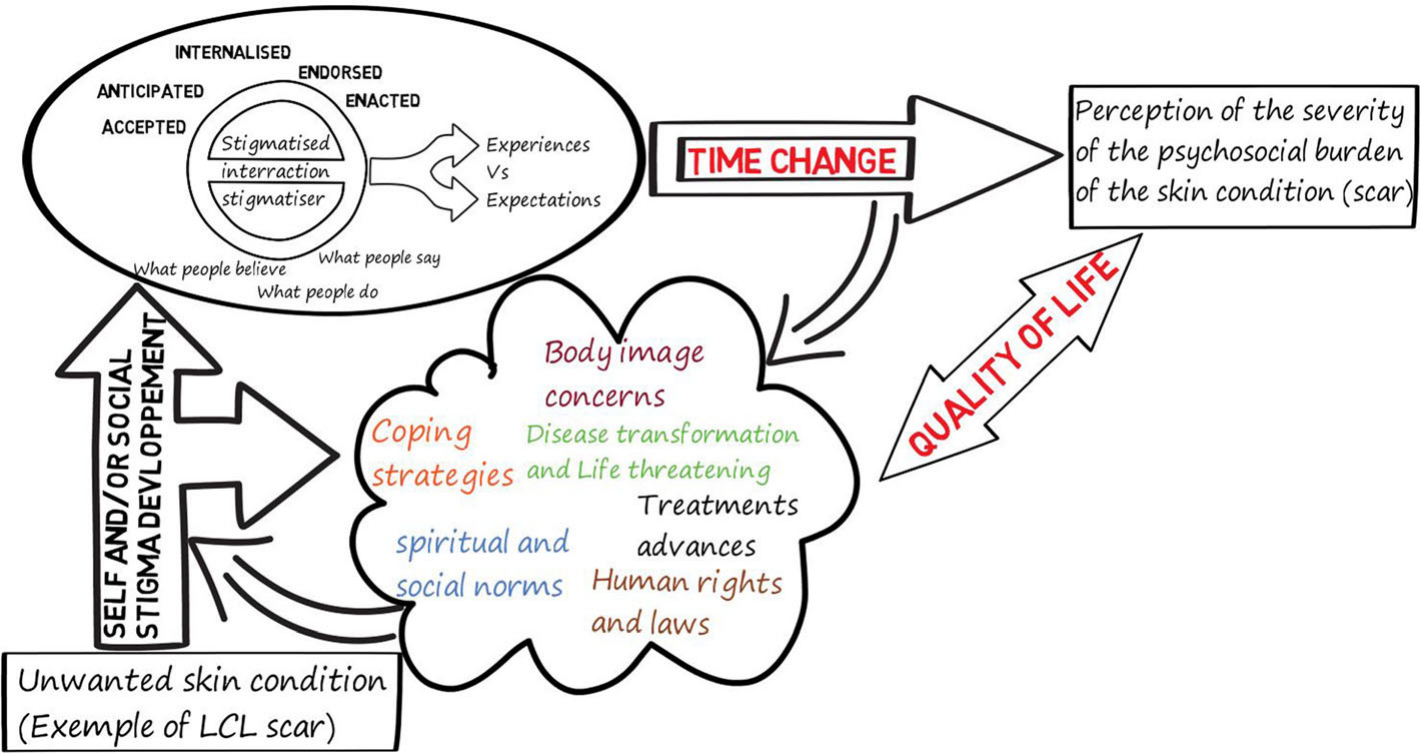
Time adjustment of stigma types and psychosocial impact of unwanted skin conditions adapted from Deacon & al [34]; Weiss [27]; and Thompson & al [62] applied to Localised cutaneous leishmaniasis (LCL)

Our review adds some new insights to the stigma theory presented previously for HIV by Deacon & al [34] and adapted for neglected tropical diseases by Weiss in 2008 [27]. We elaborated further on this model by including the reflection about body image and its distortions presented by Thompson & al [62]. We also introduced “change over time” as an important determinant for the perception of the severity and the psychosocial impact of any acquired skin disorder. Time adjustments influence the degree of self-stigma on the one hand, and on the other hand also influence the attitudes (negative or positive) society has towards the patient (Fig. 2).

The literature on burn injuries shows an adjustment over time independently of the injury size [63]. The possible change of self-perception with age is a matter of debate. For some people their self-concept could be stable but for others it could change in positive or negative ways [64, 65]; depending on the memories [66], on how emotions and memory interact and how these emotions change across lifespan [67, 68].

This scoping review can only be as strong as the studies it included, and in our case, there were some limitations. A small sample size characterized most of the included studies, and not all had appropriate control groups. E.g., the Kassi et al. [44] record is a case report presented at a symposium, telling the case of one woman with CL. The reports are not all of the same quality and are sometimes lacking detail. Reithinger et al. [42] is a short communication, describing summary results of a study that has not yet been fully published in an indexed journal. Most studies had descriptive cross-sectional quantitative survey designs with one single participant contact. Not all studies distinguished the LCL lesion from the LCL scar, though there is evidence that the effect is not the same, and that even within LCL it depends on lesion type.

Future studies should pay more attention to potential confounding factors such as the physical aspect of the lesion or scar, the socio-economic position of the person with LCL and context related factors determining the CL-related knowledge, attitudes, and practices of society [69]. Indeed, lack of knowledge about this disease and its transmission patterns can lead to collective fears, which could fuel stigmatization, rejection, and isolation such as observed in Kabul, Afghanistan. A mixed methods approach is strongly advised, as so far in many endemic areas only quantitative patient surveys were done, leaving a lot of unanswered questions about the community perception. To enhance our understanding, we recommend the development of a standard validated stigma scale for CL. Furthermore, prospective follow-up studies will more accurately describe the time-dependent changes in perception. Future qualitative research should examine stigma and quality of life components from the perspective of the patient and the health care provider and give orientations for interventions.

Our review contains some limitations. Firstly, we acknowledge that we may have missed some relevant studies because of language restrictions that excluded large parts of the world where CL is common as well (especially articles published in Chinese and Russian). Secondly, as a common limitation of scoping reviews, we did not grade the quality of evidence, to avoid loss of information, but we may have included weak evidence in this way.

## Conclusion

The literature on CL in the past 20 years claims that CL lesions, like it is the case in any other neglected infectious diseases related to poverty, influences the psychological state of those affected and can lead to a reduction in their quality of life and stigma [27, 70–75]. However, previous literature reviews assessing the burden of disease caused by leishmaniasis usually did not distinguish between the localized CL self-healing form and the other non-localised forms of CL (mucocutaneous, diffuse). Our review showed that most qualitative studies concur that LCL has indeed a negative effect on quality of life and mental health, through a process of social and self-stigmatization.

## Additional file

Additional file 1: Log book, search history, excluded articles, included articles and Data extraction labels of the main conceptual categories. (DOCX 35 kb)

## Abbreviations

BIS: Body Image Satisfaction scale
CL: Cutaneous Leishmaniasis
DALYs: Disability-Adjusted Life Years
DLQI: Dermatology Life Quality Index
DSM-IV: Diagnostic and Statistical Manual of Mental Disorders, 4th. Edition
HAD: Hospital Anxiety and Depression scale
LCL: Localized form of cutaneous leishmaniasis
MeSH: Medical Subject Headings
NTD: Neglected Tropical Disease
PRISMA: Preferred Reporting Items for Systematic Reviews and Meta-Analyses
QoL: Quality of Life
