# Supplementary material for: Psychosocial burden of localised cutaneous Leishmaniasis: a scoping review
Source: BMC Public Health. 2018 Mar 15;18:358. doi: 10.1186/s12889-018-5260-9 (PMC5855994; doi:10.1186/s12889-018-5260-9)
Supplement: Supplementary file 1 — Log book, search history, excluded articles, included articles and Data extraction labels of the main conceptual categories. (DOCX 35 kb) [file 12889_2018_5260_MOESM1_ESM.docx]

**Additional File 1 Logbook, search history, excluded articles, included articles and Data extraction labels of the main conceptual categories**

1. **Log book :**

Strategy search performed in the end of September 2016 and updated in first September 2017

| **ID** | **Database** | **Search terms** | **Number of hits** | **Without intra duplication** |
| --- | --- | --- | --- | --- |
| R01 | LILACS | Leish* AND Estigma* | 10 | 323 |
| R02 | LILACS | Leish* AND Stigma* | 45 |  |
| R03 | LILACS | Leish* AND Psychol* | 56 |  |
| R04 | LILACS | Leish* AND Psicol* | 46 |  |
| R05 | LILACS | Leish* AND scar* | 35 |  |
| R06 | LILACS | Leish* AND Cicatri* | 221 |  |
| R07 | Science Direct | Leishm*AND Stigma* | 801 | 801 |
| R08 | Cochrane Library | Leish* AND Psych* | 03 | 07 |
| R09 | Cochrane Library | Leish* AND scar* | 01 |  |
| R10 | Cochrane Library | Leishm*AND Stigma* | 03 |  |
| R11 | Cochrane Library | Cicatri* AND Stigma* | 03 |  |
| R12 | POPLINE | Leish* AND Scar* | 10 | 74 |
| R13 | POPLINE | Leish* AND stigma* | 06 |  |
| R14 | POPLINE | Leish* AND Psych* | 07 |  |
| R15 | POPLINE | Stigma* AND Scar* | 54 |  |
| R16 | PsycINFO | Leishmaniasis | 42 | 627 |
| R17 | PsycINFO | Skin Disorders OR Wounds AND Any Field: Stigma | 85 |  |
| R18 | PsycINFO | Measurement AND Any Field: stigma | 90 |  |
| R19 | PsycINFO | Measurement AND Any Field: Wounds | 64 |  |
| R20 | PsycINFO | wounds AND Index Terms: Life Changes OR Life Satisfaction OR Lifestyle Changes OR Measurement OR Mental Health OR Positive Psychology OR Quality of Life OR Wellbeing OR Work-Life Balance | 412 |  |
| R21 | PubMed | Search query listed within the article | 301 | 301 |
| R22 | Web of Knowledge | Search query listed within the article | 282 | 282 |
| R23 | Global Health | ((Leishmaniasis OR Dermal Leishmaniasis OR Cutaneous Leishmaniasis OR Mucocutaneous Leishmaniasis OR Diffuse Cutaneous Leishmaniasis NOT Visceral Leishmaniasis) AND (((Stigma) OR (Public stigma) OR (Social Stigma) OR (community stigma) OR (Enacted stigma) OR (Self-stigma) OR (Self stigma) OR (Experienced stigma) OR (Perceived stigma) OR (imagined stigma) OR (Anticipated stigma) OR (Felt stigma) OR (Self-report) OR (internalized stigma) OR (Health related stigma) OR (Gender Identity ) OR (Quality of Life) OR (Perception) OR (Rejection) OR (Discrimination)) OR ((Social problems) OR (Public opinion) OR (Social discrimination ) OR (Social isolation) OR (Social exclusion) OR (Social distance) OR (Stereotyping) OR (Social perception) OR (Social conditions) OR (Social adjustment) OR (Social behavior) OR (Social behaviour) OR (Social behavior disorders) OR (Social environment) OR (Social support) OR (Social marketing) OR (Cost of illness))) | 70 | 70 |

1. **Search history and chart flow text description**

**Step 1-** Identification of studies in 08 databases: sold with **2485** references, as shown in the table above.

**Step2-** Identification of duplicates: From the initial references treated together, we found 260 duplications for all 08 databases.

**Step 3-** Screening results: For the **2225** references screened for title or title and abstract, we excluded 2154 and kept 71.

**Step 4-** Articles included from hand searching sold with 07 additional references.

**Step 5-** After reading the full text of the 78 references defined in step 3 and, we excluded 63 references due to not corresponding to our inclusion criteria.

**Step 6-** 15 Articles included for synthesis after step 5

**Step 7-** Synthesis of the definitely included articles reported within the flow chart of this study

1. **Excluded articles after full reading text – Final Results 31th August 2017 (n=63)**

**No or too limited regarding CL stigma or CL psychosocial burden (n=33)**

Amin, T. T., et al. (2012). "Public awareness and attitudes towards cutaneous leishmaniasis in an endemic region in Saudi Arabia." Journal of the European Academy of Dermatology and Venereology 26(12): 1544-1551.

Bailey, M. S., et al. (2012). "Outbreak of zoonotic cutaneous leishmaniasis with local dissemination in Balkh, Afghanistan." J R Army Med Corps 158(3): 225-228.

Borghi, S. M., et al. (2017). "Leishmania infection: painful or painless?" Parasitol Res 116(2): 465-475.

Burki, T. (2009). "Neglected people and a neglected disease." The Lancet Infectious Diseases 9(5): 280.

Carrillo-Bonilla, L. M., et al. (2014). "Study of knowledge, attitudes, and practices related to leishmaniasis: evidence of government neglect in the Colombian Darien." Cadernos De Saude Publica 30(10): 2134-2144.

Chaves, L. F., et al. (2008). "Social Exclusion Modifies Climate and Deforestation Impacts on a Vector-Borne Disease." Plos Neglected Tropical Diseases 2(2): 8.

Costa, J. M., et al. (1998). "[Comparative study of American tegumentary leishmaniasis between childhood and teenagers from the endemic areas Buriticupu, Maranhao and Corte de Pedra, Bahia, Brazil]." Rev Soc Bras Med Trop 31(3): 279-288.

dos Reis, D. C., et al. (2006). "Health education and social representation: an experience with the control of tegumentary leishmaniasis in an endemic area in Minas Gerais, Brazil." Cad Saude Publica 22(11): 2301-2310.

Elsaie, Mohamed L., and Shady M. Ibrahim. "The effect of pulsed dye laser on cutaneous leishmaniasis and its impact on the dermatology life quality index." *Journal of Cosmetic and Laser Therapy* just-accepted (2017).

Guevara, Baílde García. "Aporte de la etnografía en el conocimiento de los códigos socioculturales de la leishmaniasis cutánea localizada en un programa de educación para la salud, en Venezuela." *Caderno de Saúde Pública* 23 (2007): 83.

González, U., et al. (2009) Interventions for American cutaneous and mucocutaneous leishmaniasis. Cochrane Database of Systematic Reviews DOI: 10.1002/14651858.CD004834.pub2

González, U., et al. (2008) Interventions for Old World cutaneous leishmaniasis. Cochrane Database of Systematic Reviews DOI: 10.1002/14651858.CD005067.pub3

Haouas, N. (2016). "Estimations of cutaneous leishmaniasis burden: a constant challenge." Lancet Infect Dis 16(5): 515-516.

Hejazi, S. H., et al. (2010). "Evaluation of knowledge, attitude and performance of the mothers of children affected by cutaneous leishmaniasis." Infectious Diseases: Research and Treatment 3: 35-40.

Kebede, N., et al. (2016). "Community knowledge, attitude and practice towards cutaneous leishmaniasis endemic area Ochello, Gamo Gofa Zone, South Ethiopia." Asian Pacific Journal of Tropical Biomedicine 6(7): 562-567.

Mathers, C. D., et al. (2007). "Measuring the burden of neglected tropical diseases: the global burden of disease framework." PLoS Negl Trop Dis 1(2): e114.

Moreira Rda, C., et al. (2002). "[knowledge level about of American tegumentary leishmaniasis (ATL) and use of alternative therapies in an endemic area in the Amazon Region in the State of Maranhao, Brazil]." Cad Saude Publica 18(1): 187-195.

Murray, C. J., et al. (2015). "Global, regional, and national disability-adjusted life years (DALYs) for 306 diseases and injuries and healthy life expectancy (HALE) for 188 countries, 1990-2013: quantifying the epidemiological transition." Lancet 386(10009): 2145-2191.

Nandha, B., et al. (2014). "Cutaneous leishmaniasis: Knowledge, attitude and practices of the inhabitants of the Kani forest tribal settlements of Tiruvananthapuram district, Kerala, India." Health Education Research 29(6): 1049-1057.

Pimenta, Denise Nacif, Anita Leandro, and Virgínia Torres Schall. "A estética do grotesco e a produção audiovisual para a educação em saúde: segregação ou empatia? O caso das leishmanioses no Brasil." (2007)

Qureshi, N. A., et al. (2016). "Prevalence of Leishmania tropica in school boys of khyber agency, FATA near Pak-Afghan border." Acta Trop 164: 90-94.

Ramdas, Sahienshadebie. "Cruel disease, cruel medicine: self-treatment of cutaneous leishmaniasis with harmful chemical substances in Suriname." *Social Science & Medicine* 75.6 (2012): 1097-1105.

Ranawaka, Ranthilaka R., and Hema S. Weerakoon. "The Quality of Life of 146 Sri Lankan Patients with Cutaneous Leishmaniasis." *Transactions of the Royal Society of Tropical Medicine and Hygiene* 97.380 (2003).

Reithinger, R., et al. (2010). "Risk Factors for Anthroponotic Cutaneous Leishmaniasis at the Household Level in Kabul, Afghanistan." Plos Neglected Tropical Diseases 4(3): 8.

Saberi, S., et al. (2012). "The knowledge, attitude, and prevention practices of students regarding cutaneous leishmaniasis in the hyperendemic region of the Shahid Babaie Airbase." Vector Borne Zoonotic Dis 12(4): 306-309.

Sarkari B, Qasem A, Shafaf MR. Knowledge, attitude, and practices related to cutaneous leishmaniasis in an endemic focus of cutaneous leishmaniasis, Southern Iran. Asian Pac J Trop Biomed. 2014;4(7):566–9.

Santos, J. B., et al. (2000). "[Socioeconomic factors and attitudes towards household prevention of American cutaneous leishmaniasis in an endemic area in Southern Bahia, Brazil]." Cad Saude Publica 16(3): 701-708.

Stewart, Carree Coffee, and William R. Brieger. "Community views on cutaneous leishmaniasis in Istalif, Afghanistan: implications for treatment and prevention." *International quarterly of community health education* 29.2 (2009): 123-142.

Turan, E., et al. (2015). "A Comparison of Demographic and Clinical Characteristics of Syrian and Turkish Patients with Cutaneous Leishmaniasis." American Journal of Tropical Medicine and Hygiene 93(3): 559-563.

Uchoa, C. M., et al. (2004). "[Health education: teaching about American tegumentary leishmaniasis]." Cad Saude Publica 20(4): 935-941.

Vazquez ML, Kroeger A, Lipowsky R, Alzate A. [Popular conceptions regarding cutaneous leishmaniasis in Colombia and their applicability in control programs]. Boletin de la Oficina Sanitaria Panamericana Pan American Sanitary Bureau. 1991;110(5):402-12. Epub 1991/05/01. PubMed PMID: 1829895.

Vlassoff, C. (1997). "The gender and tropical diseases task force of TDR: achievements and challenges." Acta Tropica 67: 173-180.

Wijeyaratne, P. M., et al. (1994). "Endemic disease and development: the leishmaniases." Acta Trop 56(4): 349-364.

**No primary study (n=16)**

Al-Kamel, Mohamed A. "Impact of leishmaniasis in women: a practical review with an update on my ISD-supported initiative to combat leishmaniasis in Yemen (ELYP)." *International Journal of Women's Dermatology* 2.3 (2016): 93-101.

Alonso, Laura Moya, and Jorge Alvar. "Enfermedades tropicales desatendidas estigmatizantes: una revisión sistemática." *Medicina Social* 5.4 (2011): 246-258.Alvar, Jorge, Sergio Yactayo, and Caryn Bern. "Leishmaniasis and poverty." *Trends in parasitology* 22.12 (2006): 552-557.

Bailey, Freddie, et al. "A new perspective on cutaneous leishmaniasis—Implications for global prevalence and burden of disease estimates." *PLOS Neglected Tropical Diseases* 11.8 (2017): e0005739.

Bern, Caryn, James H. Maguire, and Jorge Alvar. "Complexities of assessing the disease burden attributable to leishmaniasis." *PLoS Neglected Tropical Diseases* 2.10 (2008): e313.

Darío Vélez, Iván, et al. "Gender and leishmaniasis in Colombia: a redefinition of existing concepts." (1997).

Hofstraat, Karlijn, and Wim H. van Brakel. "Social stigma towards neglected tropical diseases: a systematic review." *International health* 8.suppl_1 (2016): i53-i70.

Homsi, Y., and G. Makdisi. "Leishmaniasis: a forgotten disease among neglected people." *Internet J Health* 11.2 (2010).

Karimkhani, Chante, et al. "Global burden of cutaneous leishmaniasis: a cross-sectional analysis from the Global Burden of Disease Study 2013." *The Lancet Infectious Diseases* 16.5 (2016): 584-591.

Khatami, Alireza. *Development of a Disease-Specific Instrument for Evaluation of Quality of Life in Patients with Acute Old World Cutaneous Leishmaniasis in Adult Iranian Patients: A Study Protocol*. Diss. Centre for the Public Health, 2007.

Litt, Elizabeth, Margaret C. Baker, and David Molyneux. "Neglected tropical diseases and mental health: a perspective on comorbidity." *Trends in parasitology* 28.5 (2012): 195-201.

Modabber, Farrokh, et al. "Consultative meeting to develop a strategy for treatment of cutaneous leishmaniasis. Institute Pasteur, Paris. 13–15 June, 2006." *Kinetoplastid Biology and Disease* 6.1 (2007): 3.

Mondragon-Shem, Karina, and Alvaro Acosta-Serrano. "Cutaneous leishmaniasis: the truth about the ‘flesh-eating disease’in Syria." *Trends in parasitology* 32.6 (2016): 432-435.

Okwa, O. O. "Tropical Parasitic Diseases andWomen." *Annals of African medicine* 6.4 (2007).

Okwor, Ifeoma, and Jude Uzonna. "Social and economic burden of human leishmaniasis." *The American journal of tropical medicine and hygiene* 94.3 (2016): 489-493.

.

Weigel, M. M., et al. "Gênero e leishmaniose cutânea no Equador rural: risco de doença, gravidade e conseqüências." *Doenças endêmicas: abordagens sociais, culturais e compartimentais* (2000).

Weiss, Mitchell G. "Stigma and the social burden of neglected tropical diseases." *PLoS neglected tropical diseases* 2.5 (2008): e237.

**Not a Localised CL form (n=06)**

Costa, Jackson Maurício L., et al. "Aspectos psicossociais e estigmatizantes da leishmaniose cutâneo-mucosa." *Revista da Sociedade Brasileira de Medicina Tropical* 20.2 (1987): 77-82.

Getnet, Semeneh. "The Psycho-Social Impact of Cutaneous Leishmaniasis on People Infected by the Disease." (2012).

Magalhães, Hílman M., et al. "Mudança do componente cognitivo da atitude de uma população de região endêmica do sul da Bahia diante da leishmaniose tegumentar." *Revista da Sociedade Brasileira de Medicina Tropical* 23.1 (1990): 49-52.

Toledo Jr, Antonio Carlos de Castro, et al. "Assessment of the quality of life of patients with cutaneous leishmaniasis in Belo Horizonte, Brazil, 2009–2010. A pilot study." *Transactions of The Royal Society of Tropical Medicine and Hygiene* 107.5 (2013): 335-336.

van ‘t Noordende, Anna T., et al. "Towards a toolkit for cross-neglected tropical disease morbidity and disability assessment." *International health*8.suppl_1 (2016): i71-i81.

Weigel, M. M., et al. "Cutaneous leishmaniasis in subtropical Ecuador: popular perceptions, knowledge, and treatment." (1994).

**VL or PKDL or Diffuse form of CL (n=04)**

Austveg, B. and C. Vlassoff (1993). Gender and tropical diseases: research to lift women's burden. Revised, Geneva, Switzerland, World Health Organization [WHO], Special Programme for Research and Training In Tropical Diseases, 1993.: ii, 14, [12] p.

Basher, A., et al. (2015). "A Study on Health Seeking Behaviors of Patients of Post-Kala-Azar Dermal Leishmaniasis." Biomed Res Int 2015: 314543.

Fenwick, A. (2012). "The global burden of neglected tropical diseases." Public Health 126(3): 233-236.

Rathgeber, E. M. and C. Vlassoff (1993). "Gender and tropical diseases: A new research focus." Social Science & Medicine 37(4): 513-520.

**Full text not available (n=02)**

Carrero Rangel, J. A. and R. E. Contreras Moreno (2008). "Leishmaniasis cutánea: epidemiología y aspectos psicosociales." 71-71.

Casavechia, M. T. G., et al. (2002). "A leishmaniose tegumentar sob a perspectiva do paciente: resultado de uma prática educativa." Rev. bras. anal. clin 34(4): 233-239.

**No leishmaniasis article (n=02)**

Kaur, V. (1997). "Tropical diseases and women." Clinics in Dermatology 15(1): 171-178.

van Beugen, S., et al. (2016). "Implicit stigmatization-related biases in individuals with skin conditions and their significant others." Health Psychology 35(8): 861-865.

1. **All 15 included final articles:**

1. Abazid N, Jones C, Davies CR. Knowledge, attitudes and practices about leishmaniasis among cutaneous leishmaniasis patients in Aleppo, Syrian Arab Republic. East Mediterr Health J. 2012;18(1):7-14.

2. Al-Kamel MA. Stigmata in cutaneous leishmaniasis Historical and new evidence based concepts 2017.

3. Bennis I, Thys S, Filali H, De Brouwere V, Sahibi H, Boelaert M. Psychosocial impact of scars due to cutaneous leishmaniasis on high school students in Errachidia province, Morocco. Infect Dis Poverty. 2017;6(1):46.

4. Chahed MK, Bellali H, Ben Jemaa S, Bellaj T. Psychological and Psychosocial Consequences of Zoonotic Cutaneous Leishmaniasis among Women in Tunisia: Preliminary Findings from an Exploratory Study. PLoS Negl Trop Dis. 2016;10(10):e0005090.

5. Fernando SD, Siriwardana H, Guneratne K, Rajapaksa LC. Some sociological aspects of cutaneous leishmaniasis in patients attending a tertiary referral centre in Colombo, Sri Lanka. International Health. 2010;2(1):69-74.

6. Handjani F, Kalafi A. Impact of dermatological diseases on family members of the patients using Family Dermatology Life Quality Index: a preliminary study in Iran. Iranian Journal of Dermatology. 2013;16(4):128-31.

7. Kassi M, Kassi M, Afghan AK, Rehman R, Kasi PM. Marring Leishmaniasis: The Stigmatization and the Impact of Cutaneous Leishmaniasis in Pakistan and Afghanistan. Plos Neglect Trop Dis. 2008;2(10):3.

8. Nilforoushzadeh MA, Roohafza HR, Jaffary F, Khatun M. Comparison of quality of life in women suffering from Cutaneous leishmaniasis treated with topical and systemic glucantime along with psychiatric consultation compared with the group without psychiatric consultation. Journal of Isfahan Medical School. 2012;29(172):unpaginated.

09. Ramdas S, van der Geest S, Schallig H. Nuancing stigma through ethnography: the case of cutaneous leishmaniasis in Suriname. Social Science & Medicine. 2016;151:139-46.

10. Reithinger R, Aadil K, Kolaczinski J, Mohsen M, Hami S. Social impact of leishmaniasis, Afghanistan. Emerg Infect Dis. 2005;11(4):634-6.

11. Reyburn H. SOCIAL AND PSYCHOLOGICAL CONSEQUENCES OF CUTANEOUS LEISHMANIASIS IN KABUL, AFGHANISTAN. HEALTHNETINTERNATIONAL IN PARTNERSHIP WITH NORWEGIAN CHURCH AID. 2000.

12. Simsek Z, Ak D, Altindag A, Günes M. Prevalence and predictors of mental disorders among women in Sanliurfa, Southeastern Turkey. J Public Health (Oxf). 2008;30(4):487-93.

13. Turan E, Kandemir H, Yesilova Y, Ekinci S, Tanrikulu O, Kandemir SB, et al. Assessment of psychiatric morbidity and quality of life in children and adolescents with cutaneous leishmaniasis and their parents. Postep Dermatol Alergol. 2015;32(5):344-8.

14. Vares B, Mohseni M, Heshmatkhah A, Farjzadeh S, Safizadeh H, Shamsi-Meymandi S, et al. Quality of Life in Patients with Cutaneous Leishmaniasis. Arch Iran Med. 2013;16(8):474-7.

15. Yanik M, Gurel MS, Simsek Z, Kati M. The psychological impact of cutaneous leishmaniasis. Clin Exp Dermatol. 2004;29(5):464-7.

1. **Data extraction labels of the main conceptual categories used in NVivo 11**

Country of the study

Author names, year, journal

Study design (cross sectional, case control …) and type (Quantitative, Qualitative)

Population experiencing CL (characteristics)

Sample size (number of participants, gender, age)

Ratio Men/women CL affected

Cutaneous Leishmaniasis species

Dermatological condition involved (CL lesions, CL scars)

Types of stigma (Cite the exact names and types used by those authors)

Types of psychological consequences

Reasons for stigma related to the dermatological condition

Notion of time change of the perception of stigma

Notion of time change of the perception of psychological consequences

Comparison between CL lesions and CL scars

Comparison between CL and other NTD

Comparison between CL and other (No NTD) dermatological diseases

Coping strategies targeting CL outcomes

Measurement scale of stigma

Measurement of psychological consequences

Main finding
